# Association of dietary flavonoid intakes with prevalence of chronic respiratory diseases in adults

**DOI:** 10.1186/s12967-024-04949-7

**Published:** 2024-02-26

**Authors:** Runmiao Wu, Xu Zhu, Gongchang Guan, Qianwei Cui, Ling Zhu, Yujie Xing, Jingsha Zhao

**Affiliations:** 1 Department of Respiratory and Critical Care Medicine, Shaanxi Provincial People’s Hospital, Xi’an, 710000 Shaanxi China; 2 Department of Cardiology, The First Affiliated Hospital of Nanjing Medical University, Jiangsu Province Hospital, Nanjing, 210029 China; 3 Department of Cardiology, Shaanxi Provincial People’s Hospital, 256 Youyi West Road, Xi’an, 710000 Shaanxi China; 4 Department of Cardiology, The Third Affiliated Hospital of Xi’an Jiaotong University, Xi’an, 710000 Shaanxi China; 5 Department of Intensive Care Unit, The Third People’s Hospital of Chengdu, 82 Qinglong Road, Chengdu, Sichuan China

**Keywords:** Flavonoids, Isoflavones, Anthocyanidins, Flavan-3-ols, Flavanones, Flavones, Flavonols, Chronic respiratory diseases

## Abstract

**Background and aims:**

Flavonoids are a class of secondary plant metabolites that have been shown to have multiple health benefits, including antioxidant and anti-inflammatory. This study was to explore the association between dietary flavonoid consumption and the prevalence of chronic respiratory diseases (CRDs) in adults.

**Methods and results:**

The six main types of flavonoids, including isoflavones, anthocyanidins, flavan-3-ols, flavanones, flavones, and flavonols, were obtained from the National Health and Nutrition Examination Survey (NHANES) 2007–2010 and 2017–2018 by the two 24-h recall interviews. The prevalence of CRDs, including asthma, emphysema, and chronic bronchitis, was determined through a self-administered questionnaire. The analysis included 15,753 participants aged 18 years or older who had completed a diet history interview. After adjustment for potential confounders, the inverse link was found with total flavonoids, anthocyanidins, flavanones, and flavones, with an OR (95%CI) of 0.86 (0.75–0.98), 0.84 (0.72–0.97), 0.80(0.69–0.92), and 0.85(0.73–0.98) for the highest group compared to the lowest group. WQS regression revealed that the mixture of flavonoids was negatively linked with the prevalence of CRDs (OR = 0.88 [0.82–0.95], *P* < 0.01), and the largest effect was mainly from flavanones (weight = 0.41). In addition, we found that flavonoid intake was negatively linked with inflammatory markers, and systemic inflammation significantly mediated the associations of flavonoids with CRDs, with a mediation rate of 12.64% for CRP (*P* < 0.01).

**Conclusion:**

Higher flavonoid intake was related with a lower prevalence of CRDs in adults, and this relationship may be mediated through systemic inflammation.

**Supplementary Information:**

The online version contains supplementary material available at 10.1186/s12967-024-04949-7.

## Introduction

Chronic Respiratory Diseases (CRDs) encompass a spectrum of enduring respiratory ailments, notably including asthma, emphysema, and chronic bronchitis [1]. These conditions constitute a substantial global public health challenge, characterized by their elevated prevalence, considerable disease burden, and profound ramifications for the quality of life of afflicted individuals [2, 3]. According the Global Burden of Diseases, Injuries, and Risk Factors Study (GBD), CRDs are responsible for approximately 9 million deaths annually, accounting for 7% of all deaths worldwide [1]. The incidence of CRDs is on the rise, affecting an estimated 544 million individuals worldwide [4, 5]. Furthermore, CRDs rank among the leading causes of disability, inflicting symptoms such as persistent coughing, wheezing, and chest constriction, which significantly impede daily activities and overall well-being [6]. Additionally, CRDs frequently co-occur with comorbidities, including cardiovascular disease, diabetes, and depression, further amplifying the burden on both patients and healthcare systems [7–9]. The multifaceted risk factors associated with CRDs encompass environmental variables such as air pollution, genetic predisposition, respiratory infections, and tobacco exposure [10–12]. Dietary patterns and other lifestyle factors also play pivotal roles in the onset and progression of CRDs [13, 14].

Flavonoids represent a class of naturally occurring compounds characterized by a flavone scaffold with diverse functional groups, resulting in a wide array of chemical structures [15]. Principal dietary sources of flavonoids encompass fruits like berries, citrus fruits, and apples, as well as vegetables such as onions, broccoli, and kale [16]. Furthermore, tea and wine serve as notable reservoirs of flavonoids [17]. Flavonoids have been associated with a plethora of health benefits, prominently featuring anti-inflammatory, antioxidant, and immunomodulatory properties [18–20]. These attributes have the potential to mitigate or ameliorate a spectrum of maladies, including cardiovascular diseases, malignancies, and dermatological conditions [21–23]. Elevated consumption of anthocyanins and flavan-3-ols has been linked to a diminished risk of cardiovascular disease, while flavanols and flavones have demonstrated associations with an elevated risk of coronary heart disease [24]. Additional investigations have evidenced that increased intake of flavonols, flavones, and isoflavones is correlated with a reduced incidence of breast, ovarian, and endometrial cancers [25]. Moreover, flavonoids have exhibited neuroprotective qualities, potentially mitigating the risk of neurodegenerative disorders [26]. Additionally, they may foster gastrointestinal health by promoting the proliferation of beneficial gut microbiota while mitigating inflammation [27]. In summation, the consumption of a diverse array of flavonoid-rich foods holds the potential to bolster overall health and reduce the risk of chronic diseases.

A pivotal facet of flavonoid bioactivity is their capacity to modulate inflammation and oxidative stress within the body [16]. Inflammation constitutes a natural immune response deployed by the body to ward off injury or infection; nevertheless, excessive or persistent inflammation can engender a wide spectrum of diseases [28]. Flavonoids have evinced promising anti-inflammatory attributes through the inhibition of pro-inflammatory enzymes and cytokines, attenuation of reactive oxygen species (ROS) generation, and the interception of inflammation-related signaling pathways [29]. While numerous studies have corroborated the association between flavonoids and health outcomes, relatively scant research has probed the link between total flavonoid intake or specific flavonoid subclasses and CRDs, an ailment intimately entwined with inflammation. Our study was conceived to leverage data from the extensive and representative population-based 2007–2010 National Health and Nutrition Examination Survey (NHANES) to scrutinize the associations between flavonoid consumption and CRDs. Additionally, we endeavored to explore the role of inflammatory responses within this nexus.

## Materials and methods

### Study population

The National Health and Nutrition Examination Survey (NHANES) is a program conducted by the Centers for Disease Control and Prevention (CDC) in the US [30]. It is a survey with national coverage that gathers information on the general population's medical and dietary status in the US. Its research content covers a wide range of health-related topics and has had a significant impact on disease research and public health policies in the US. NHANES is composed of two main components: a health interview and a physical examination. Public health policies and actions targeted at lowering the burden of disease in the US population have been informed by NHANES data. The study protocol received approval from the Research Ethics Review Board of the National Center, and all participants provided informed consent.

We obtained the data from the NHANES 2007–2010 and 2017–2018. Participants with missing data on flavonoid intake were excluded (n = 3,715). Participants with age < 20 and missing data on CRDs were excluded (n = 10,301). Finally, after removing pregnant women (n = 171), a total of 15,753 participants were included in the analysis (Additional file 1: Figure S1).

### Assessment of flavonoids and CRDs

NHANES utilizes dietary recall interviews to estimate the intake levels of dietary flavonoids. The trained interviewers collect detailed information about the types and quantities of foods and beverages consumed by participants over 24 h. This information is then used to calculate the flavonoid content of each food item using a comprehensive flavonoid database. To ensure accurate estimation of flavonoid intake, NHANES utilizes the US Department of Agriculture Food and Nutrient Database for Dietary Studies (FNDDS)’s flavonoid database, which contains comprehensive information on the flavonoid content of various foods and beverages. Our study estimated the intake of total flavonoids, isoflavones, anthocyanidins, flavan-3-ols, flavanones, flavones, and flavonols in all foods and beverages. The total flavonoid intake was derived from the sum of 29 individual flavonoids (Additional file 1: Table S1). The detailed estimation process was described in previous study [31].

NHANES utilizes questionnaire-based assessments to evaluate CRDs. Participants are asked to complete a questionnaire that collects information on their respiratory health, including symptoms, medical history, and medication use. This questionnaire helps identify individuals who may have chronic respiratory conditions, such as asthma, emphysema, or chronic bronchitis [32].

### Assessment of Covariates

Information regarding participants' baseline data was collected, including age, sex, race, education level, and total energy intake. Poverty Income Ratio (PIR) is a measure of income relative to the poverty threshold [33]. Participants were asked to provide information about their household income, family size, and poverty guidelines are used to determine the PIR, and then categorized into different income groups, such as low income (≤ 1.0), middle income (1.1–3.0), and high income (> 3.0). We evaluated smoking status through self-reported information collected during the survey [34]. Participants were asked questions about their current and past smoking habits, including whether they currently smoke, have ever smoked, or are former smokers. We obtained information on the prevalence of metabolic syndrome and supplement use among participants using self-reported questionnaires.

### Statistical analysis

The major sampling units, sample weights, and strata were all taken into account throughout the data analysis to give credible national estimates, as recommended by the National Center for Health Statistics. The weighted analyses were carried out using the R package "survey". Individuals' starting characteristics are expressed as numbers with percentages, and categorical variables were compared using the chi-square test. We analyzed the concentration and the distribution of different types of flavonoids. Spearman correlation analysis was used to calculate the correlation coefficients among all flavonoids. All analyses were performed using R (version 4.2.0) and *P*-values less than 0.05 were considered statistically significant.

The flavonoids intake levels were log-transformed to normalize their distributions as continuous variable and divided into three groups as categorical variable. Among the six types of flavonoid intakes, 58.0%, 35.3%, and 38.4% of the population reported zero intake of isoflavones, anthocyanidins, and flavanones, respectively. Consequently, individuals with no intake of isoflavones, anthocyanidins, and flavanones were placed in the first group, while those with non-zero intake were divided equally into two groups. Due to the fact that the proportion of individuals with zero intake of flavan-3-ols, flavones, and flavonols was less than 33.3%, the participants were evenly divided into three groups.

Multiple logistic regression was used to analyze the link between flavonoid intake (continuous or categorical variable) and the prevalence of CRDs in the US population. Potential non-linear links between flavonoid intake and prevalence of CRDs were analysed using restricted cubic spline (RCS) regression with 10th, 50th and 90th percentile as nodes. We conducted comprehensive stratified analyses across various subgroups, focusing on age groups (< 60 years versus ≥ 60 years), gender (male versus female), and racial categories (non-Hispanic Whites versus others). Weighted quantile sum (WQS) regression was performed using “gWQS” package to assess the mixed effects of multiple exposure variables on a given outcome [35, 36]. It was used to assess the negative association of the mixture of six dietary flavonoids intakes with the prevalence of CRDs and its components.

We also analyzed the associations of dietary total flavonoid intake levels with inflammatory markers (C-reactive protein [CRP], white blood cell count [WBC], neutrophil, lymphocyte, monocyte, and red blood Cell distribution width [RDW]) among adults in 2007–2018 NHANES. The mediated effect of flavonoid intake on CRDs through inflammation marker was calculated. The mediating effect of the inflammation marker between dietary flavonoids and CRDs was analysed using the R package “mediation”. To execute mediation analyses, three distinct pathways were established: the path from the exposure variable to the mediator, and from the mediator to the outcome variable (representing indirect effects), and the path from the exposure variable to the outcome variable (representing the direct effect) [37]. The summation of the direct and mediated (indirect) effects constitutes the entirety of the total effect. To quantify the extent of mediation, the mediated effect was divided by the total effect, yielding the percentage of mediated effects. The statistical significance of the mediation analyses was ascertained employing the Bootstrapping method.

## Results

### Participant characteristics at baseline

A total of 15,753 adults (2,842 participants with CRDs) took part in this study, including 2,193 asthma participants, 969 chronic bronchitis individuals, and 364 emphysema patients (Table 1). Of the study participants, 35.91% were over 59 years of age, 49.25% were male, 44.32% were non-Hispanic white, and 49.74% reported supplement use. Participants with CRDs were more likely to be older (> 59 years), female, non-Hispanic white, lower income, current smokers, and higher prevalence of metabolic syndrome, compared to participants without CRDs (all *P* < 0.01).

**Table 1:** Survey-weighted, sociodemographic and health status characteristics of adult NHANES 2007–2010 and 2017–2018 participants with available data

Characteristics	Total (n = 15,753)	Chronic respiratory diseases		*P* value
		No (n = 12,911)	Yes (n = 2842)	
Age, years				0.01
< 40	4945 (31.39)	4067 (36.08)	878 (35.67)	
40–59	5151 (32.7)	4259 (38.14)	892 (35.02)	
> 59	5657 (35.91)	4585 (25.78)	1072 (29.31)	
Sex, %				< 0.01
Female	7995 (50.75)	6393 (49.67)	1602 (58.00)	
Male	7758 (49.25)	6518 (50.33)	1240 (42.00)	
Race/ethnicity, %				< 0.01
Mexican American	2566 (16.29)	2314 (9.19)	252 (4.72)	
Other Hispanic	1598 (10.14)	1324 (5.61)	274 (5.03)	
Non-Hispanic White	6982 (44.32)	5542 (66.83)	1440 (70.07)	
Non-Hispanic Black	3251 (20.64)	2600 (10.87)	651 (13.03)	
Other race	1356 (8.61)	1131 (7.50)	225 (7.15)	
Education level, %				0.09
Below high school	4170 (26.47)	3427 (16.13)	743 (17.66)	
High school	3763 (23.89)	3021 (25.00)	742 (26.88)	
Above high school	7820 (49.64)	6463 (58.87)	1357 (55.46)	
Family PIR, %				< 0.01
≤ 1.0	3300 (20.95)	2582 (13.12)	718 (17.98)	
1.1–3.0	6820 (43.29)	5568 (35.58)	1252 (37.86)	
> 3.0	5633 (35.76)	4761 (51.30)	872 (44.15)	
Smoking status, %				< 0.01
Never smoker	8547 (54.26)	7329 (57.43)	1218 (45.13)	
Former smoker	3917 (24.87)	3097 (24.11)	820 (28.60)	
Current smoker	3289 (20.88)	2485 (18.46)	804 (26.28)	
Total energy intakes, kcal/day				0.01
Tertile 1	5251 (33.33)	4205 (28.47)	1046 (32.50)	
Tertile 2	5256 (33.37)	4386 (34.50)	870 (31.01)	
Tertile 3	5246 (33.3)	4320 (37.03)	926 (36.49)	
Metabolic syndrome, %				< 0.01
No	9398 (59.67)	7846 (64.48)	1552 (59.15)	
Yes	6353 (40.33)	5064 (35.52)	1289 (40.85)	
Supplement use, %				0.35
No	7918 (50.26)	6520 (46.97)	1398 (48.15)	
Yes	7835 (49.74)	6391 (53.03)	1444 (51.85)	
Self-reported asthma, %				< 0.01
No	13,560 (86.08)	12,911 (100.00)	649 (21.85)	
Yes	2193 (13.92)	0 (0.00)	2193 (78.15)	
Self-reported chronic bronchitis, %				< 0.01
No	14,784 (93.85)	12,911 (100.00)	1873 (67.55)	
Yes	969 (6.15)	0 (0.00)	969 (32.45)	
Self-reported emphysema, %				< 0.01
No	15,389 (97.69)	12,911 (100.00)	2478 (90.01)	
Yes	364 (2.31)	0 (0.00)	364 (9.99)	

PIR, poverty income ratio. Categorical variables are presented as numbers (percentages). Sampling weights were applied for calculation of demographic descriptive statistics. N reflect the study sample while percentages reflect the survey-weighted data

### Distributions and concentrations of dietary flavonoid intake

Additional file 1: Table S2 listed the distribution and concentration of dietary flavonoid intakes among adults. The mean total flavonoids, isoflavones, anthocyanidins, flavan-3-ols, flavanones, flavones, flavonols was 207.52, 1.75, 11.64, 161.63, 13.59, 0.87, and 18.04 mg/day, respectively. Additional file 1: Figure S2 showed the Spearman correlation coefficients among dietary flavonoid. The Spearman correlation between flavonoids ranged from weak (r = 0.06 for isoflavones and flavan-3-ols) to high (r = 0.67 for flavan-3-ols and total flavonols).

### Association between dietary flavonoid intake and the prevalence of CRDs

**Fig. 1:**
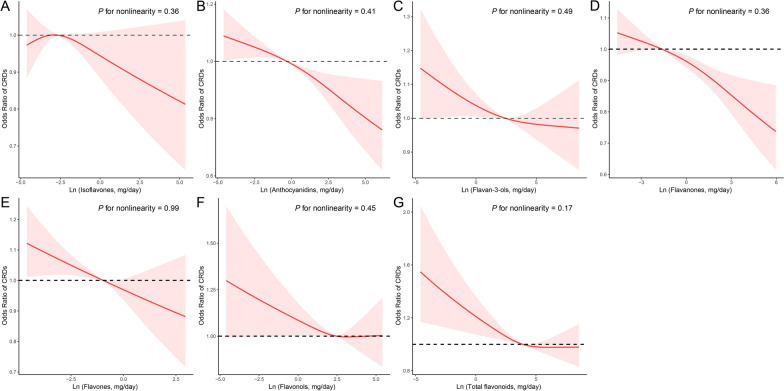
The exposure–response associations of dietary flavonoid intake and chronic respiratory diseases (CRDs) by restricted cubic spline (RCS) model in adults. Model was adjusted for age (< 40, 40–59, or > 59), sex (male or female), race/ethnicity (Mexican American, Other Hispanic, Non-Hispanic White, Non-Hispanic Black or Other), education level (below high school, high school, or above high school), family poverty income ratio (≤ 1.0, 1.1–3.0, or > 3.0), smoking status (never smoker, former smoker, or current smoker), energy intake levels (in tertiles), metabolic syndrome (yes or no), and supplement use (yes or no)

Multiple logistic regression analysis of the link between dietary flavonoid intake and the prevalence of CRDs was shown in Table 2. In crude model, we found that higher intakes of total flavonoids and its components were significantly linked with a lower prevalence of CRDs (both continuous and categorical variables). After adjustment for potential confounders, continuous flavonoid intake was found to be negatively associated with the prevalence of CRDs (OR [95% CI] 0.97 [0.94,0.99] for total flavonoids; 0.97 [0.95–0.99] for anthocyanidins; 0.97[0.96–0.99] for flavanones; and 0.97[0.94,1.00] for flavones). Similarly, the inverse link was found with total flavonoids, anthocyanidins, flavanones, and flavones, with an OR of 0.86 (95% CI: 0.75–0.98), 0.84 (95% CI: 0.72–0.97), 0.80(95% CI: 0.69–0.92), and 0.85(95% CI: 0.73–0.98) for the highest group compared to the lowest group. RCS regression showed that all types of flavonoids were non-linearly related to the prevalence of CRDs (*P* for non-linearity > 0.05) in Fig. 1. We also investigated the link between flavonoid intake and specific CRDs prevalence (Additional file 1: Table S2).

**Table 2:** ORs (95% CIs) of the prevalence of chronic respiratory diseases (CRDs) according to dietary flavonoid intake levels (mg/day) among adults in NHANES 2007–2010 and 2017–2018

	Continous flavonoid intakes		Category of flavonoid intakes			
	OR (95% CI)	*P* value	Group 1	Group 2	Group 3	*P*_trend_
Isoflavones						
Crude	0.97(0.95,1.00)	0.04	Ref (1.00)	0.87(0.76,1.01)	0.86(0.74,1.00)	0.03
Model 1	0.98(0.95,1.00)	0.07	Ref (1.00)	0.88(0.76,1.02)	0.90(0.77,1.05)	0.10
Model 2	0.99(0.96,1.02)	0.41	Ref (1.00)	0.92(0.79,1.07)	0.96(0.82,1.13)	0.48
Anthocyanidins						
Crude	0.96(0.94,0.98)	< 0.01	Ref (1.00)	0.86(0.75,0.98)	0.76(0.66,0.88)	< 0.01
Model 1	0.96(0.94,0.98)	< 0.01	Ref (1.00)	0.86(0.75,0.98)	0.73(0.63,0.85)	< 0.01
Model 2	0.97(0.95,0.99)	0.01	Ref (1.00)	0.93(0.80,1.07)	0.84(0.72,0.97)	0.02
Flavan-3-ols						
Crude	0.98(0.96,1.00)	0.02	Ref (1.00)	0.85(0.74,0.99)	0.83(0.73,0.93)	< 0.01
Model 1	0.98(0.96,0.99)	0.01	Ref (1.00)	0.86(0.74,1.00)	0.81(0.72,0.92)	< 0.01
Model 2	0.99(0.97,1.01)	0.20	Ref (1.00)	0.92(0.78,1.07)	0.88(0.77,1.01)	0.07
Flavanones						
Crude	0.96(0.94,0.98)	< 0.01	Ref (1.00)	0.87(0.74,1.02)	0.73(0.63,0.84)	< 0.01
Model 1	0.96(0.94,0.98)	< 0.01	Ref (1.00)	0.86(0.73,1.01)	0.72(0.62,0.83)	< 0.01
Model 2	0.97(0.96,0.99)	< 0.01	Ref (1.00)	0.94(0.79,1.11)	0.80(0.69,0.92)	< 0.01
Flavones						
Crude	0.94(0.92,0.96)	< 0.01	Ref (1.00)	0.78(0.68,0.90)	0.74(0.65,0.84)	< 0.01
Model 1	0.95(0.92,0.97)	< 0.01	Ref (1.00)	0.78(0.68,0.90)	0.76(0.67,0.87)	< 0.01
Model 2	0.97(0.94,1.00)	0.04	Ref (1.00)	0.84(0.73,0.97)	0.85(0.73,0.98)	0.03
Flavonols						
Crude	0.95(0.91,0.98)	0.01	Ref (1.00)	0.86(0.73,1.01)	0.84(0.73,0.96)	0.01
Model 1	0.96(0.92,1.00)	0.03	Ref (1.00)	0.89(0.75,1.04)	0.88(0.76,1.01)	0.06
Model 2	0.98(0.93,1.02)	0.28	Ref (1.00)	0.93(0.79,1.10)	0.92(0.80,1.07)	0.29
Total flavonoids						
Crude	0.95(0.93,0.98)	< 0.01	Ref (1.00)	0.80(0.69,0.93)	0.79(0.70,0.90)	< 0.01
Model 1	0.95(0.93,0.98)	< 0.01	Ref (1.00)	0.81(0.70,0.94)	0.78(0.69,0.89)	< 0.01
Model 2	0.97(0.94,0.99)	0.05	Ref (1.00)	0.87(0.75,1.02)	0.86(0.75,0.98)	0.03

Model 1 was adjusted for age (< 40, 40–59, or > 59), sex (male or female), and race/ethnicity (Mexican American, Other Hispanic, Non-Hispanic White, Non-Hispanic Black or Other); Model 2 was adjusted as model 1 plus education level (below high school, high school, or above high school), family poverty income ratio (≤ 1.0, 1.1–3.0, or > 3.0), smoking status (never smoker, former smoker, or current smoker), energy intake levels (in tertiles), metabolic syndrome (yes or no), and supplement use (yes or no)

### Stratified Analysis

Detailed stratified analyses delineate the complex associations between dietary flavonoid intake and CRDs prevalence. Additional file 1: Table S3 details age-based variation (< 60 or ≥ 60 years) in CRDs prevalence relative to flavonoid consumption. Additional file 1: Table S4 investigates gender-specific differences (male versus female) in flavonoid intake's impact on CRDs prevalence. Additional file 1: Table S5 examines racial disparities (non-Hispanic White versus other races), elucidating the interplay between dietary habits and CRDs prevalence in different racial groups. The results of our stratified analyses indicate that the interaction effects between dietary flavonoid intake and demographic factors such as age, sex, and race on CRDs prevalence are not statistically significant. This suggests that the relationship between dietary flavonoid compounds and CRDs does not vary significantly across different ages, genders, and racial groups.

### Effect of the mixture of flavonoids on CRDs

**Fig. 2:**
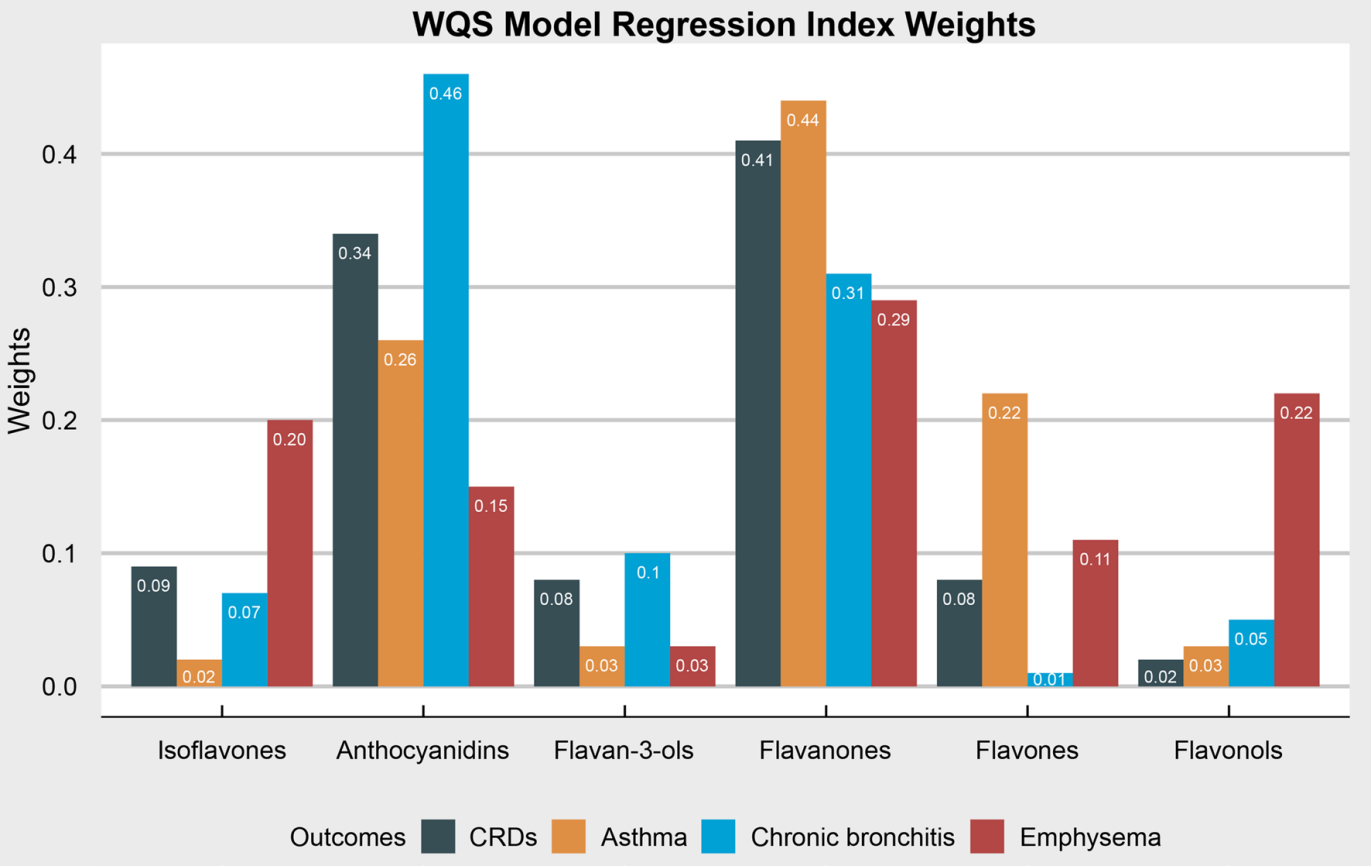
Weights from weighted quantile sum regression (WQS) for the mixture of flavonoids in relation to the prevalence of chronic respiratory diseases (CRDs) in adults. Model was adjusted for age (< 40, 40–59, or > 59), sex (male or female), race/ethnicity (Mexican American, Other Hispanic, Non-Hispanic White, Non-Hispanic Black or Other), education level (below high school, high school, or above high school), family poverty income ratio (≤ 1.0, 1.1–3.0, or > 3.0), smoking status (never smoker, former smoker, or current smoker), energy intake levels (in tertiles), metabolic syndrome (yes or no), and supplement use (yes or no)

The WQS regression was applied to assess the link between flavonoid mixture and the prevalence of CRDs (Table 3). We found that the mixture of flavonoids was negatively linked with the prevalence of CRDs (OR [95% CI] 0.88 [0.82–0.95], *P* < 0.01), asthma (OR [95% CI] 0.92 [0.85–0.99], *P* = 0.02), chronic bronchitis (OR [95% CI] 0.88 [0.78–0.98], *P* = 0.03), and emphysema (OR [95% CI] 0.70 [0.55–0.89], *P* < 0.01). The largest effect was mainly from flavanones (weight = 0.41 for CRDs, 0.44 for asthma, and 0.29 for emphysema) and anthocyanidins (weight = 0.46 for CRDs) (Fig. 2).

**Table 3:** WQS regression model to assess the negative association of the mixture of six dietary flavonoids intakes with the prevalence of chronic respiratory diseases (CRDs) and its components

Outcomes	OR	95%CI	*P* value
CRDS	0.88	0.82–0.95	< 0.01
Asthma	0.92	0.85–0.99	0.02
Chronic bronchitis	0.88	0.78–0.98	0.03
Emphysema	0.70	0.55–0.89	< 0.01

OR, odds ratio; CI, confidence interval; WQS, weighted quantile sum. WQS regression model was adjusted for age (< 40, 40–59, or > 59), sex (male or female), race/ethnicity (Mexican American, Other Hispanic, Non-Hispanic White, Non-Hispanic Black or Other), education level (below high school, high school, or above high school), family poverty income ratio (≤ 1.0, 1.1–3.0, or > 3.0), smoking status (never smoker, former smoker, or current smoker), energy intake levels (in tertiles), metabolic syndrome (yes or no), and supplement use (yes or no)

### Mediation effects of inflammatory markers

Furthermore, we analyzed the associations of dietary total flavonoid intake levels with inflammatory markers in adults. We found that both continuous and categorical flavonoid intake were negatively linked with inflammatory markers (such as CRP, WBC, neutrophil, monocyte, and RDW) (Table 4). The CRP, WBC, neutrophil, and RDW levels mediated the associations of flavonoids with CRDs by 12.64%, 5.42%, 5.01%, and 8.34%, respectively (Table 5).

**Table 4:** Multiple linear regression associations of dietary total flavonoid intake levels with inflammatory markers among adults in NHANES 2007–2010 and 2017–2018

	Continous flavonoid intakes		Category of flavonoid intakes			
	β (95% CI)	*P* value	β	β (95% CI)	β (95% CI)	*P*_trend_
CRP	− 0.04 (− 0.05, − 0.02)	< 0.01	Ref (0.00)	− 0.16 (− 0.23, − 0.09)	− 0.17 (− 0.23, − 0.11)	< 0.01
WBC	− 0.01 (− 0.01, − 0.00)	< 0.01	Ref (0.00)	− 0.03 (− 0.04, − 0.01)	− 0.04 (− 0.05, − 0.02)	< 0.01
NEU	− 0.01 (− 0.01, − 0.00)	< 0.01	Ref (0.00)	− 0.04 (− 0.06, − 0.02)	− 0.05 (− 0.07, − 0.03)	< 0.01
LYM	0.00 (− 0.01, 0.00)	0.29	Ref (0.00)	− 0.02 (− 0.04, 0.00)	− 0.02 (− 0.04, 0.00)	0.05
MON	− 0.01 (− 0.01, − 0.00)	0.01	Ref (0.00)	− 0.02 (− 0.04, − 0.01)	− 0.02 (− 0.04, − 0.01)	0.01
RDW	− 0.03 (− 0.04, − 0.02)	< 0.01	Ref (0.00)	− 0.16 (− 0.23, − 0.10)	− 0.14 (− 0.19, − 0.09)	< 0.01

CI, confidence interval; CRP, C-reactive protein; WBC, white blood cell count; NEU, neutrophil count; LYM, lymphocyte count; MON, monocyte count; RDW, red blood Cell distribution width. Model was adjusted as age (< 40, 40–59, or > 59), sex (male or female), race/ethnicity (Mexican American, Other Hispanic, Non-Hispanic White, Non-Hispanic Black or Other), education level (below high school, high school, or above high school), family poverty income ratio (≤ 1.0, 1.1–3.0, or > 3.0), smoking status (never smoker, former smoker, or current smoker), energy intake levels (in tertiles), metabolic syndrome (yes or no), and supplement use (yes or no)

**Table 5:** The mediation effects of inflammatory markers on the association of dietary total flavonoid intake levels with the prevalence of chronic respiratory diseases (CRDs) among adults in NHANES 2007–2010 and 2017–2018

Mediators	Indirect effects	Direct effects	Mediated proportion (%)	*P* value
	β (95%CI)	β (95%CI)		
CRP	− 0.0023 (− 0.0034, − 0.0014)	− 0.0156 (− 0.0300, − 0.0026)	12.64%	0.01
WBC	− 0.0010 (− 0.0018, − 0.0003)	− 0.0164 (− 0.0302, − 0.0035)	5.42%	0.02
NEU	− 0.0009 (− 0.0017, − 0.0003)	− 0.0170 (− 0.0312, − 0.0021)	5.01%	0.02
LYM	0.0001 (− 0.0001, 0.0004)	− 0.0179 (− 0.0327, − 0.0034)	0.43%	0.42
MON	− 0.0004 (− 0.0009, 0.0001)	− 0.0173 (− 0.0326, − 0.0031)	1.93%	0.12
RDW	− 0.0015 (− 0.0025, − 0.0008)	− 0.0161 (− 0.0286, − 0.0013)	8.34%	0.03

CI, confidence interval; CRP, C-reactive protein; WBC, white blood cell count; NEU, neutrophil count; LYM, lymphocyte count; MON, monocyte count; RDW, red blood Cell distribution width. Model was adjusted as age (< 40, 40–59, or > 59), sex (male or female), race/ethnicity (Mexican American, Other Hispanic, Non-Hispanic White, Non-Hispanic Black or Other), education level (below high school, high school, or above high school), family poverty income ratio (≤ 1.0, 1.1–3.0, or > 3.0), smoking status (never smoker, former smoker, or current smoker), energy intake levels (in tertiles), metabolic syndrome (yes or no), and supplement use (yes or no)

## Discussion

We analyzed the link between dietary flavonoid intake and CRDs prevalence in adults from a large cohort in the 2007–2010 and 2017–2018 NHANES. We found that higher intakes of total flavonoids and its components (anthocyanidins, flavanones, and flavones) were linked with lower CRDs prevalence in adults. Furthermore, WQS regression also revealed that the mixture of flavonoids was negatively linked with the prevalence of CRDs, and the largest effect was mainly from flavanones. In addition, we found a negative correlation between flavonoid intake and inflammatory markers, and systemic inflammation partially mediated the associations of flavonoids with CRDs.

Flavonoids, a class of phytochemical compounds derived from plants, have garnered extensive scrutiny due to their potential health-promoting attributes [38]. These compounds have been linked to a diminished risk of chronic ailments owing to their antioxidative and anti-inflammatory properties [39]. In populations characterized by elevated total flavonoid consumption, a consistent reduction in the risk of mortality attributable to cardiovascular disease, ischemic heart disease, and cerebrovascular disease has been observed [40]. Multifactorial analyses have corroborated significant negative associations between anthocyanin and flavanone intake and the risk of hyperuricemia [41]. Notably, Xie et al. discerned that flavonoid intake, along with its subcategories, exhibited an inverse relationship with hepatic steatosis and fibrosis [42]. Similarly, Moslehi et al. established that augmented flavonoid consumption is conducive to mitigating the risk of metabolically unhealthy phenotypes among overweight and obese adults [43]. In a study encompassing two extensive U.S. cohorts, heightened flavonoid consumption, particularly of anthocyanins and flavan-3-ols, correlated with a reduced mortality risk among individuals with Parkinson's disease [44]. Moreover, in a study involving 2,856 adults, each incremental unit of dietary total flavonol intake was associated with a lower prevalence of age-related macular degeneration (AMD) [45]. In the context of our investigation, it is pertinent to underscore that total flavonoids, anthocyanidins, flavanones, and flavones were all associated with a diminished prevalence of CRDs.

A multitude of investigations exploring the nexus between flavonoids and specific CRDs have yielded insights suggesting the potential efficacy of flavonoids in both the prevention and treatment of these conditions. For instance, a study originating from the Netherlands disclosed independent associations between total catechins, flavonols, and flavonoid intake with three symptoms of COPD [46]. These findings underscore the potential benefits of a high intake of catechins and solid fruits for individuals afflicted with COPD, with the negative correlation between flavonoid intake and COPD being particularly pronounced among smokers [47]. Furthermore, Borghi et al. furnished evidence indicating the utility of flavonoids as active agents in the management of asthma [48]. A randomized controlled trial corroborated the efficacy of purple passion fruit peel extract, rich in bioflavonoids, in ameliorating clinical symptoms among asthma patients [49]. Mattioli et al. found that flavanones may reduce risk of non-allergic rhinitis [50]. Importantly, our investigation not only affirms the association between specific flavonoids and CRDs but also underscores the potential advantages of a flavonoid mixture in mitigating the prevalence of CRDs, particularly with regard to flavanones. Additionally, we have demonstrated that the inflammatory marker CRP may play a mediating role in the aforementioned associations.

CRDs are distinguished by persistent airway inflammation and heightened oxidative stress [51]. Flavonoids have been demonstrated to intricately regulate these pathophysiological processes through a multifaceted array of mechanisms. Firstly, flavonoids function as free radical scavengers, mitigating oxidative stress within the airways [52]. This attribute holds particular significance since oxidative stress possesses the capacity to inflict cellular damage and incite inflammation, thereby contributing to the initiation and progression of chronic respiratory diseases [53]. Secondly, flavonoids possess the capability to modulate the immune response in the airway milieu [54]. In cases of asthma, flavonoids exhibit inhibitory effects on the production of pro-inflammatory cytokines and chemokines, thereby engendering an anti-inflammatory milieu [55]. Thirdly, flavonoids elicit enhancements in pulmonary function by facilitating bronchodilation and ameliorating airway hyperresponsiveness [56]. The aggregate of evidence suggests that the mechanisms underpinning the protective effects of flavonoids in the context of chronic respiratory diseases are intricate and multifactorial. Nevertheless, their antioxidative and anti-inflammatory attributes, coupled with their immunomodulatory potential and capacity to ameliorate lung function, bestow upon them a promising candidacy for therapeutic intervention in the realm of chronic respiratory diseases.

In examining the relationship between dietary flavonoids and CRDs, it is necessary to consider the role of the gut microbiome [57–61]. Flavonoids, a diverse group of polyphenolic compounds, are metabolized by gut microbiota, resulting in the production of various biologically active metabolites [57, 60]. These metabolites can exert systemic effects, potentially influencing respiratory health through several mechanisms. Metabolites produced from flavonoid degradation by gut bacteria might have anti-inflammatory properties [61]. They may modulate inflammatory pathways, which are often implicated in chronic respiratory diseases like asthma and COPD. By reducing systemic inflammation, these metabolites could potentially mitigate the severity of respiratory conditions [62]. Moreover, flavonoids impact the gut barrier function [62]. A healthy gut barrier is crucial in maintaining systemic immune homeostasis. Flavonoids can enhance barrier integrity, thereby preventing the translocation of pro-inflammatory molecules from the gut to the rest of the body, including the respiratory system [62]. Lastly, the gut-lung axis, a bidirectional communication pathway between the gut and the lungs, is an emerging area of interest [63]. The gut microbiome's role in this axis is critical, as it can influence lung health both directly and indirectly through immune modulation and the production of microbial metabolites [62]. In summary, the interaction between dietary flavonoids and the gut microbiome represents a complex and significant area of research, particularly in the context of respiratory health [61]. This underscores the importance of considering the gut microbiome in dietary strategies aimed at preventing or managing chronic respiratory diseases [59].

In prior studies, one population-based multi-case–control study analyzed the associations between chronic respiratory diseases and intakes of total flavonoids and their major subclasses, revealing a significant correlation between increased flavanone intake and reduced risk of non-allergic rhinitis [50]. Another study from the Danish Diet, Cancer, and Health research examined the relationships between total flavonoids, flavonoid subclasses, and major flavonoid compounds with COPD incidence, concluding that higher total flavonoid intakes were linked to a 20% lower risk of COPD [47]. In contrast to existing studies, we conducted a comprehensive analysis encompassing a broader spectrum of flavonoids, including isoflavones, anthocyanidins, flavan-3-ols, flavanones, flavones, and flavonols, and assessed their association with various CRDs, such as asthma, emphysema, and chronic bronchitis. While previous studies focused on specific flavonoids or diseases like COPD and non-allergic rhinitis, we leveraged the extensive dataset from the NHANES, which covers a large and diverse population. This choice enhances the robustness and generalizability of our findings. Moreover, our study introduced a novel dimension by exploring the mediating role of systemic inflammation in the relationship between flavonoid intake and CRDs. This aspect was not the primary focus of the aforementioned studies. This approach provides valuable insights into the potential mechanistic pathways through which flavonoids may exert their effects on respiratory health, enhancing the depth of understanding in the field. Methodologically, we employed WQS regression analysis and meticulously adjusted for numerous potential confounders, providing a more nuanced and reliable understanding of the flavonoids-CRDs relationship. This innovative approach allows for the exploration of the combined effects of flavonoid compounds as a mixture on respiratory diseases [36]. By doing so, it sidesteps the challenges posed by covariance and non-linearity among highly correlated dietary flavonoids. This methodological innovation broadens the scope of the investigation and offers a nuanced perspective on the relationship between flavonoids and CRDs.

Nevertheless, there exist several limitations that warrant consideration. Firstly, it is imperative to acknowledge that while this research may establish an association between dietary flavonoid intakes and CRDs, it cannot definitively establish causality. The inherent limitations of observational studies restrict the capacity to draw causal inferences, and further interventional research is needed to substantiate any causal relationships and determine optimal intake levels of flavonoids for respiratory health. Secondly, the reliance on self-reported measures for both dietary intake and disease prevalence introduces potential sources of bias. Recall bias and reporting bias may influence the accuracy of the results, as individuals may not consistently recall their dietary habits or respiratory health status with precision. However, studies have also reported that this method has demonstrated its effectiveness in accurately capturing dietary element intake, minimizing measurement error and enhancing the precision of the dietary exposure variable [64, 65]. Thirdly, it is crucial to recognize that this study predominantly focused on a United States population. Therefore, the generalizability of the findings to other populations must be approached with caution.

## Conclusion

This study found that higher intakes of flavonoids (anthocyanidins, flavanones, and flavones) were linked with a lower prevalence of CRDs in adults. Flavanones were found to have the strongest inverse association with CRDs. Furthermore, systemic inflammation partially mediated the associations of flavonoids with CRDs. These findings suggest that flavonoid-rich diets may be beneficial for the prevention and management of CRDs.

### Supplementary Information

**Additional file 1:** **Figure S1**. Eligible participants in the evaluation of the influence between dietary flavonoid intakes and the prevalence of chronic respiratory diseases in the general adult population.** Figure S2**.** Table S1.** Distributions and concentrations of dietary flavonoid intakes (mg/day) among adults in NHANES 2007–2010 and 2017–2018.** Table S2.** ORs (95% CIs) of the prevalence of specific chronic respiratory diseases (CRDs) according to dietary flavonoid intake levels (mg/day) among adults in NHANES 2007–2010 and 2017–2018. Pairwise Pearson correlation coefficients among dietary flavonoids in adults.** Table S3. **Stratified analyses of the prevalence of chronic respiratory diseases (CRDs) according to dietary flavonoid intake levels (mg/day) by age (<60, or ≥60 years) in NHANES 2007–2010 and 2017–2018.** Table S4. **Stratified analyses of the prevalence of chronic respiratory diseases (CRDs) according to dietary flavonoid intake levels (mg/day) by sex (male, or female) in NHANES 2007–2010 and 2017–2018.** Table S5. **Stratified analyses of the prevalence of chronic respiratory diseases (CRDs) according to dietary flavonoid intake levels (mg/day) by race (non-Hispanic White, or other race) in NHANES 2007–2010 and 2017–2018.

## Data Availability

NHANES data described in this manuscript are available at: https://wwwn.cdc.gov/nchs/nhanes/.
