# Supplementary material for: Association of dietary flavonoid intakes with prevalence of chronic respiratory diseases in adults
Source: J Transl Med. 2024 Feb 26;22:205. doi: 10.1186/s12967-024-04949-7 (PMC10898189; doi:10.1186/s12967-024-04949-7)
Supplement: Supplementary file 1 — Additional file 1: Figure S1. Eligible participants in the evaluation of the influence between dietary flavonoid intakes and the prevalence of chronic respiratory diseases in the general adult population. Figure S2. Table S1. Distributions and concentrations of dietary flavonoid intakes (mg/day) among adults in NHANES 2007–2010 and 2017–2018. Table S2. ORs (95% CIs) of the prevalence of specific chronic respiratory diseases (CRDs) according to dietary flavonoid intake levels (mg/day) among adults in NHANES 2007–2010 and 2017–2018. Pairwise Pearson correlation coefficients among dietary flavonoids in adults. Table S3. Stratified analyses of the prevalence of chronic respiratory diseases (CRDs) according to dietary flavonoid intake levels (mg/day) by age (<60, or ≥60 years) in NHANES 2007–2010 and 2017–2018. Table S4. Stratified analyses of the prevalence of chronic respiratory diseases (CRDs) according to dietary flavonoid intake levels (mg/day) by sex (male, or female) in NHANES 2007–2010 and 2017–2018. Table S5. Stratified analyses of the prevalence of chronic respiratory diseases (CRDs) according to dietary flavonoid intake levels (mg/day) by race (non-Hispanic White, or other race) in NHANES 2007–2010 and 2017–2018. [file 12967_2024_4949_MOESM1_ESM.docx]

**Additional Materials**

**Association of Dietary Flavonoid intakes with Prevalence of Chronic Respiratory Diseases in Adults**

Runmiao Wu; Xu Zhu; Gongchang Guan; Qianwei Cui; Ling Zhu; Yujie Xing; Jingsha Zhao

**
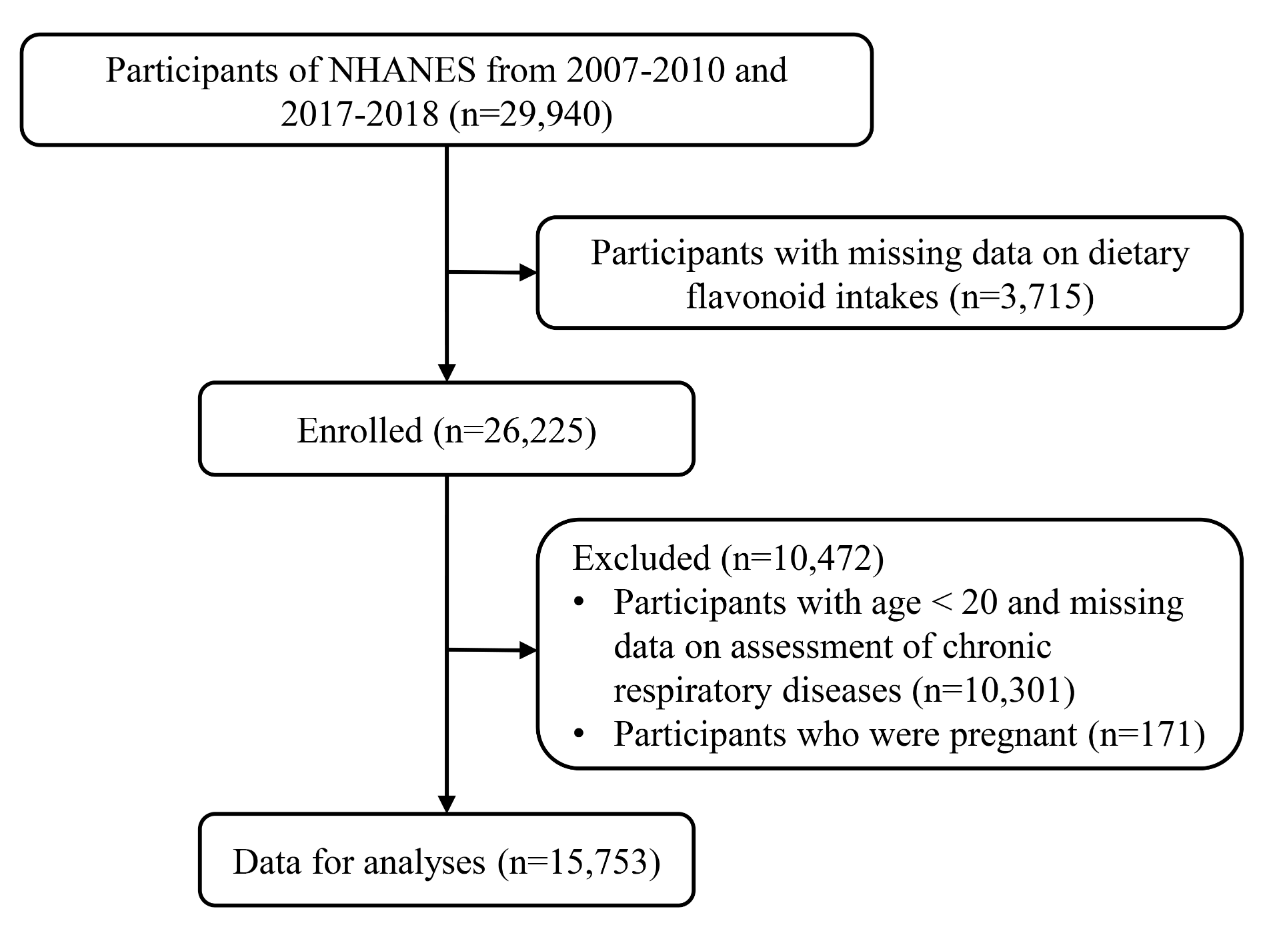
**

**Additional file 1: Figure S1**. Eligible participants in the evaluation of the influence between dietary flavonoid intakes and the prevalence of chronic respiratory diseases in the general adult population.

**Additional file 1: Table S1.** Distributions and concentrations of dietary flavonoid intakes (mg/day) among adults in NHANES 2007–2010 and 2017–2018.

| Flavonoid class | Mean | 5^th^ | 25^th^ | 50^th^ | 75^th^ | 95^th^ |
| --- | --- | --- | --- | --- | --- | --- |
| ***Isoflavones*** |  |  |  |  |  |  |
| Daidzein | 0.68 | 0.00 | 0.00 | 0.00 | 0.01 | 2.35 |
| Genistein | 0.94 | 0.00 | 0.00 | 0.00 | 0.03 | 3.05 |
| Glycitein | 0.13 | 0.00 | 0.00 | 0.00 | 0.00 | 0.39 |
| Total Isoflavones | 1.75 | 0.00 | 0.00 | 0.00 | 0.04 | 5.72 |
| ***Anthocyanidins*** |  |  |  |  |  |  |
| Cyanidin | 2.25 | 0.00 | 0.00 | 0.21 | 1.59 | 6.89 |
| Delphinidin | 1.39 | 0.00 | 0.00 | 0.00 | 0.16 | 5.62 |
| Malvidin | 4.11 | 0.00 | 0.00 | 0.00 | 0.15 | 27.70 |
| Pelargonidin | 1.38 | 0.00 | 0.00 | 0.00 | 0.13 | 8.70 |
| Peonidin | 1.60 | 0.00 | 0.00 | 0.00 | 0.37 | 4.09 |
| Petunidin | 0.91 | 0.00 | 0.00 | 0.00 | 0.05 | 4.12 |
| Total anthocyanidins | 11.64 | 0.00 | 0.00 | 0.72 | 6.21 | 60.68 |
| ***Flavan-3-ols*** |  |  |  |  |  |  |
| (-)-Epicatechin* | 9.59 | 0.00 | 0.48 | 3.73 | 13.76 | 35.12 |
| (-)-Epicatechin 3-gallate* | 10.02 | 0.00 | 0.00 | 0.00 | 0.44 | 58.13 |
| (-)-Epigallocatechin* | 15.46 | 0.00 | 0.03 | 0.31 | 1.98 | 87.64 |
| (-)-Epigallocatechin 3-gallate* | 26.75 | 0.00 | 0.00 | 0.00 | 0.86 | 155.69 |
| (+)-Catechin* | 7.45 | 0.00 | 0.89 | 4.28 | 10.17 | 25.19 |
| (+)-Gallocatechin* | 1.61 | 0.00 | 0.00 | 0.00 | 0.72 | 9.20 |
| Theaflavin | 1.48 | 0.00 | 0.00 | 0.00 | 0.00 | 9.48 |
| Theaflavin-3,3'-digallate | 1.63 | 0.00 | 0.00 | 0.00 | 0.00 | 10.46 |
| Theaflavin-3'-gallate | 1.38 | 0.00 | 0.00 | 0.00 | 0.00 | 8.94 |
| Theaflavin-3-gallate | 1.17 | 0.00 | 0.00 | 0.00 | 0.00 | 7.46 |
| Thearubigins | 85.10 | 0.00 | 0.00 | 0.00 | 0.00 | 509.95 |
| Total Flavan-3-ols | 161.63 | 0.00 | 2.61 | 11.24 | 53.36 | 894.92 |
| ***Flavanones*** |  |  |  |  |  |  |
| Eriodictyol | 0.22 | 0.00 | 0.00 | 0.00 | 0.03 | 0.97 |
| Hesperetin | 9.71 | 0.00 | 0.00 | 0.00 | 2.74 | 56.08 |
| Naringenin | 3.66 | 0.00 | 0.00 | 0.14 | 1.22 | 20.29 |
| Total Flavanones | 13.59 | 0.00 | 0.00 | 0.20 | 5.24 | 73.80 |
| ***Flavones*** |  |  |  |  |  |  |
| Apigenin | 0.20 | 0.00 | 0.00 | 0.04 | 0.18 | 0.79 |
| Luteolin | 0.68 | 0.00 | 0.04 | 0.26 | 0.80 | 2.72 |
| Total Flavones | 0.87 | 0.00 | 0.07 | 0.38 | 1.06 | 3.19 |
| ***Flavonols*** |  |  |  |  |  |  |
| Isorhamnetin | 0.88 | 0.00 | 0.00 | 0.34 | 1.09 | 3.34 |
| Kaempferol | 4.62 | 0.02 | 0.49 | 1.99 | 5.88 | 17.41 |
| Myricetin | 1.46 | 0.01 | 0.18 | 0.49 | 1.56 | 5.99 |
| Quercetin | 11.08 | 0.55 | 3.26 | 7.54 | 14.71 | 33.39 |
| Total Flavonols | 18.04 | 1.07 | 5.52 | 12.09 | 23.37 | 53.39 |
| ***Total sum of all 29 flavonoids*** | 207.52 | 3.14 | 16.75 | 48.66 | 172.03 | 981.50 |

5^th^, 5th percentile; 25^th^, 25th percentile; 50^th^, 50th percentile; 75^th^, 75th percentile; 95^th^, 95th percentile.


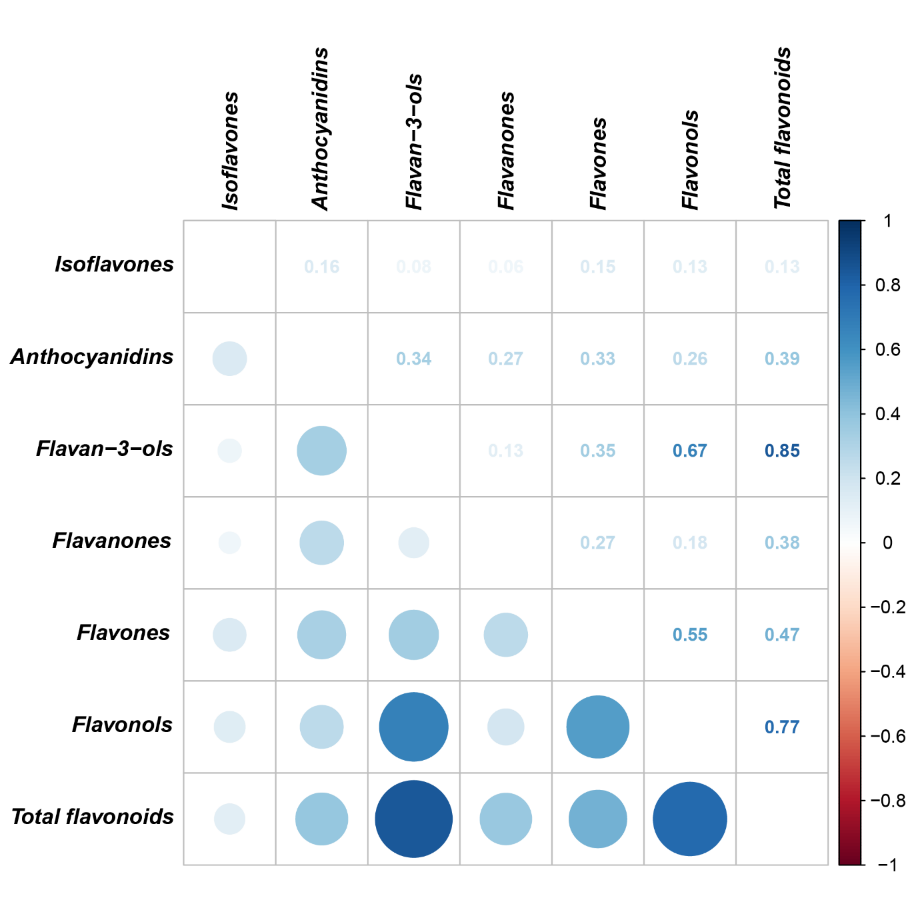


**Additional file 1: Figure S2**. Pairwise Pearson correlation coefficients among dietary flavonoids in adults.

**Table S2.** ORs (95% CIs) of the prevalence of specific chronic respiratory diseases (CRDs) according to dietary flavonoid intake levels (mg/day) among adults in NHANES 2007–2010 and 2017–2018.

| Flavonoids | Continous flavonoid intakes | |  | Category of flavonoid intakes | | |  |
| --- | --- | --- | --- | --- | --- | --- | --- |
|  | OR (95% CI) | *P* value |  | Group 1 | Group 2 | Group 3 | *P* _trend_ |
| ***Asthma*** |  |  |  |  |  |  |  |
| Isoflavones | 1.00(0.96,1.03) | 0.77 |  | Ref (1.00) | 0.86(0.72,1.04) | 0.97(0.80,1.18) | 0.54 |
| Anthocyanidins | 0.98(0.96,1.00) | 0.04 |  | Ref (1.00) | 0.94(0.80,1.10) | 0.90(0.78,1.04) | 0.14 |
| Flavan-3-ols | 0.99(0.97,1.01) | 0.37 |  | Ref (1.00) | 1.01(0.85,1.21) | 0.95(0.82,1.10) | 0.44 |
| Flavanones | 0.98(0.96,1.00) | 0.02 |  | Ref (1.00) | 0.90(0.74,1.09) | 0.83(0.71,0.97) | 0.02 |
| Flavones | 0.97(0.93,1.00) | 0.07 |  | Ref (1.00) | 0.81(0.68,0.97) | 0.86(0.72,1.03) | 0.10 |
| Flavonols | 0.97(0.93,1.01) | 0.17 |  | Ref (1.00) | 0.87(0.74,1.02) | 0.91(0.78,1.05) | 0.21 |
| Total flavonoids | 0.98(0.95,1.00) | 0.09 |  | Ref (1.00) | 0.95(0.80,1.14) | 0.92(0.80,1.07) | 0.27 |
| ***Chronic bronchitis*** | |  |  |  |  |  |  |
| Isoflavones | 0.98(0.94,1.02) | 0.35 |  | Ref (1.00) | 1.00(0.76,1.33) | 0.99(0.76,1.29) | 0.95 |
| Anthocyanidins | 0.97(0.94,1.00) | 0.07 |  | Ref (1.00) | 0.98(0.77,1.24) | 0.78(0.60,1.02) | 0.06 |
| Flavan-3-ols | 1.01(0.97,1.04) | 0.76 |  | Ref (1.00) | 0.84(0.67,1.05) | 0.98(0.77,1.24) | 0.89 |
| Flavanones | 0.98(0.95,1.01) | 0.16 |  | Ref (1.00) | 1.11(0.87,1.41) | 0.77(0.57,1.04) | 0.11 |
| Flavones | 0.99(0.95,1.04) | 0.75 |  | Ref (1.00) | 0.91(0.73,1.13) | 0.91(0.71,1.17) | 0.45 |
| Flavonols | 1.02(0.95,1.10) | 0.50 |  | Ref (1.00) | 1.01(0.77,1.31) | 1.04(0.85,1.28) | 0.67 |
| Total flavonoids | 1.00(0.95,1.05) | 0.99 |  | Ref (1.00) | 0.84(0.65,1.10) | 0.94(0.73,1.20) | 0.62 |
| ***Emphysema*** |  |  |  |  |  |  |  |
| Isoflavones | 0.94(0.86, 1.03) | 0.16 |  | Ref (1.00) | 0.96(0.62, 1.48) | 0.67(0.44, 1.02) | 0.07 |
| Anthocyanidins | 0.95(0.89, 1.02) | 0.15 |  | Ref (1.00) | 0.90(0.61, 1.32) | 0.78(0.48, 1.27) | 0.30 |
| Flavan-3-ols | 0.98(0.92, 1.04) | 0.51 |  | Ref (1.00) | 1.09(0.74, 1.61) | 0.71(0.48, 1.03) | 0.07 |
| Flavanones | 0.96(0.90, 1.01) | 0.10 |  | Ref (1.00) | 0.94(0.63, 1.40) | 0.70(0.45, 1.10) | 0.13 |
| Flavones | 0.92(0.86, 0.99) | 0.03 |  | Ref (1.00) | 0.78(0.57, 1.07) | 0.73(0.46, 1.17) | 0.16 |
| Flavonols | 0.95(0.87, 1.03) | 0.20 |  | Ref (1.00) | 1.02(0.66, 1.57) | 0.81(0.59, 1.10) | 0.17 |
| Total flavonoids | 0.98(0.91, 1.06) | 0.59 |  | Ref (1.00) | 0.78(0.52, 1.16) | 0.71(0.51, 1.00) | 0.05 |

Model 1 was adjusted for age (<40, 40-59, or > 59), sex (male or female), and race/ethnicity (Mexican American, Other Hispanic, Non-Hispanic White, Non-Hispanic Black or Other); Model 2 was adjusted as model 1 plus education level (below high school, high school, or above high school), family poverty income ratio (≤1.0, 1.1–3.0, or >3.0), smoking status (never smoker, former smoker, or current smoker), energy intake levels (in tertiles), metabolic syndrome (yes or no), and supplement use (yes or no).

**Additional file 1: Table S3.** Stratified analyses of the prevalence of chronic respiratory diseases (CRDs) according to dietary flavonoid intake levels (mg/day) by age (<60, or ≥60 years) in NHANES 2007–2010 and 2017–2018.

|  | Age <60 (n=10096) | | |  | Age ≥60 (n=5657) | | |  | *P-int* |
| --- | --- | --- | --- | --- | --- | --- | --- | --- | --- |
|  | Group 1 | Group 2 | Group 3 |  | Group 1 | Group 2 | Group 3 |  |  |
| Isoflavones | Ref (1.00) | 0.89(0.75,1.06) | 0.92(0.74,1.14) |  | Ref (1.00) | 0.95(0.76,1.19) | 0.96(0.76,1.23) |  | 0.83 |
| Anthocyanidins | Ref (1.00) | 0.91(0.75,1.11) | 0.81(0.67,0.98) |  | Ref (1.00) | 0.82(0.63,1.06) | 0.75(0.58,0.99) |  | 0.75 |
| Flavan-3-ols | Ref (1.00) | 0.89(0.73,1.10) | 0.87(0.75,1.02) |  | Ref (1.00) | 0.89(0.73,1.09) | 0.79(0.61,1.03) |  | 0.71 |
| Flavanones | Ref (1.00) | 0.86(0.73,1.02) | 0.78(0.65,0.93) |  | Ref (1.00) | 1.06(0.83,1.35) | 0.78(0.60,1.01) |  | 0.18 |
| Flavones | Ref (1.00) | 0.79(0.66,0.93) | 0.76(0.63,0.92) |  | Ref (1.00) | 0.89(0.70,1.13) | 0.96(0.79,1.17) |  | 0.29 |
| Flavonols | Ref (1.00) | 0.91(0.77,1.08) | 0.88(0.77,1.02) |  | Ref (1.00) | 0.92(0.70,1.21) | 0.98(0.77,1.24) |  | 0.73 |
| Total flavonoids | Ref (1.00) | 0.85(0.72,1.00) | 0.85(0.73,0.99) |  | Ref (1.00) | 0.82(0.63,1.06) | 0.76(0.59,0.97) |  | 0.70 |

Analyses were adjusted for covariates age (<40, 40-59, or > 59), sex (male or female), and race/ethnicity (Mexican American, Other Hispanic, Non-Hispanic White, Non-Hispanic Black or Other); Model 2 was adjusted as model 1 plus education level (below high school, high school, or above high school), family poverty income ratio (≤1.0, 1.1–3.0, or >3.0), smoking status (never smoker, former smoker, or current smoker), energy intake levels (in tertiles), metabolic syndrome (yes or no), and supplement use (yes or no) when they were not the strata variables. *p-int*, p for interaction.

**Additional file 1: Table S4.** Stratified analyses of the prevalence of chronic respiratory diseases (CRDs) according to dietary flavonoid intake levels (mg/day) by sex (male, or female) in NHANES 2007–2010 and 2017–2018.

|  | Male (n=7995) | | |  | Female (n=7758) | | |  | *P-int* |
| --- | --- | --- | --- | --- | --- | --- | --- | --- | --- |
|  | Group 1 | Group 2 | Group 3 |  | Group 1 | Group 2 | Group 3 |  |  |
| Isoflavones | Ref (1.00) | 0.94(0.74,1.21) | 0.93(0.72,1.20) |  | Ref (1.00) | 0.89(0.73,1.07) | 0.94(0.80,1.09) |  | 0.90 |
| Anthocyanidins | Ref (1.00) | 0.85(0.69,1.04) | 0.77(0.59,0.99) |  | Ref (1.00) | 0.93(0.76,1.15) | 0.83(0.70,0.98) |  | 0.79 |
| Flavan-3-ols | Ref (1.00) | 0.75(0.56,1.01) | 0.67(0.51,0.88) |  | Ref (1.00) | 1.04(0.86,1.26) | 1.03(0.84,1.26) |  | 0.06 |
| Flavanones | Ref (1.00) | 1.11(0.87,1.40) | 0.89(0.71,1.12) |  | Ref (1.00) | 0.79(0.63,0.98) | 0.70(0.57,0.85) |  | 0.07 |
| Flavones | Ref (1.00) | 0.86(0.65,1.12) | 0.77(0.61,0.97) |  | Ref (1.00) | 0.79(0.65,0.96) | 0.86(0.70,1.05) |  | 0.44 |
| Flavonols | Ref (1.00) | 0.76(0.57,1.00) | 0.81(0.62,1.06) |  | Ref (1.00) | 1.05(0.86,1.28) | 0.99(0.82,1.20) |  | 0.09 |
| Total flavonoids | Ref (1.00) | 0.73(0.58,0.92) | 0.72(0.57,0.91) |  | Ref (1.00) | 0.96(0.79,1.17) | 0.92(0.78,1.09) |  | 0.10 |

Analyses were adjusted for covariates age (<40, 40-59, or > 59), sex (male or female), and race/ethnicity (Mexican American, Other Hispanic, Non-Hispanic White, Non-Hispanic Black or Other); Model 2 was adjusted as model 1 plus education level (below high school, high school, or above high school), family poverty income ratio (≤1.0, 1.1–3.0, or >3.0), smoking status (never smoker, former smoker, or current smoker), energy intake levels (in tertiles), metabolic syndrome (yes or no), and supplement use (yes or no) when they were not the strata variables. *p-int*, p for interaction.

**Additional file 1: Table S5.** Stratified analyses of the prevalence of chronic respiratory diseases (CRDs) according to dietary flavonoid intake levels (mg/day) by race (non-Hispanic White, or other race) in NHANES 2007–2010 and 2017–2018.

|  | Non-Hispanic White (n=8771) | | |  | Other race (n=6982) | | |  | *P-int* |
| --- | --- | --- | --- | --- | --- | --- | --- | --- | --- |
|  | Group 1 | Group 2 | Group 3 |  | Group 1 | Group 2 | Group 3 |  |  |
| Isoflavones | Ref (1.00) | 0.88(0.72,1.09) | 0.79(0.67,0.93) |  | Ref (1.00) | 0.90(0.75,1.09) | 0.98(0.79,1.22) |  | 0.44 |
| Anthocyanidins | Ref (1.00) | 0.78(0.66,0.92) | 0.65(0.56,0.76) |  | Ref (1.00) | 0.91(0.75,1.10) | 0.85(0.70,1.03) |  | 0.57 |
| Flavan-3-ols | Ref (1.00) | 0.89(0.76,1.03) | 0.93(0.76,1.12) |  | Ref (1.00) | 0.89(0.74,1.08) | 0.83(0.71,0.98) |  | 0.17 |
| Flavanones | Ref (1.00) | 0.94(0.77,1.13) | 0.68(0.55,0.83) |  | Ref (1.00) | 0.89(0.72,1.09) | 0.81(0.67,0.99) |  | 0.31 |
| Flavones | Ref (1.00) | 0.74(0.60,0.91) | 0.63(0.52,0.76) |  | Ref (1.00) | 0.83(0.71,0.98) | 0.88(0.74,1.05) |  | 0.12 |
| Flavonols | Ref (1.00) | 0.97(0.80,1.17) | 0.86(0.71,1.04) |  | Ref (1.00) | 0.88(0.71,1.09) | 0.92(0.77,1.10) |  | 0.49 |
| Total flavonoids | Ref (1.00) | 0.80(0.69,0.94) | 0.81(0.65,0.99) |  | Ref (1.00) | 0.86(0.70,1.05) | 0.83(0.71,0.98) |  | 0.73 |

Analyses were adjusted for covariates age (<40, 40-59, or > 59), sex (male or female), and race/ethnicity (Mexican American, Other Hispanic, Non-Hispanic White, Non-Hispanic Black or Other); Model 2 was adjusted as model 1 plus education level (below high school, high school, or above high school), family poverty income ratio (≤1.0, 1.1–3.0, or >3.0), smoking status (never smoker, former smoker, or current smoker), energy intake levels (in tertiles), metabolic syndrome (yes or no), and supplement use (yes or no) when they were not the strata variables. *p-int*, p for interaction.
